# Contrast induced nephropathy in urology

**DOI:** 10.4103/0970-1591.57904

**Published:** 2009

**Authors:** Viji Samuel Thomson, Kumar Narayanan, J. Chandra Singh

**Affiliations:** Department of Cardiology, Christian Medical College, Vellore, India; 1 Department of Urology, Christian Medical College, Vellore, India

**Keywords:** Fenoldopam, isoosmolar agents, N-acetylcysteine, radiocontrast-induced nephropathy, ultrafiltration

## Abstract

Intravenous contrast agents have a distinct role in urological imaging: to study precise anatomical delineation, vascularity, and to assess the function of the renal unit. Contrast induced nephropathy (CIN) is a known adverse effect of intravenous contrast administration. The literature on incidence, pathophysiology, clinical features, and current preventive strategies available for CIN relevant to urologists was reviewed. A search of the PubMed database was done using the keywords nephropathy and media, prevention and control or prevention Contrast media (explode), all adverse effects, and kidney diseases (explode). An online search of the EMBASE database for the time ranging from 1977 to February 2009 was performed using the keywords ionic contrast medium, adverse drug reaction, major or controlled clinical study, human, nephrotoxicity, and kidney disease. Current publications and data most relevant to urologists were examined. CIN was the third most common cause of hospital-acquired renal failure. The incidence is less common with intravenous contrast administration as compared with intra-arterial administration. The pathogenesis of contrast mediated nephropathy is due to a combination of toxic injury to renal tubules and medullary ischemic injury mediated by reactive oxygen species. CIN most commonly manifests as a nonoliguric and asymptomatic transient decline in renal function. Patients who developed CIN were found to have increased mortality, longer hospital stay, and complicated clinical course. An overview of risk factors and risk prediction score for prognostication of CIN are elaborated. Preventive strategies including choice of contrast agents, maximum tolerated dose, role of hydration, hydration regime, etc. are discussed. The role of N- acetyl cysteine, Theophylline, Fenoldapam, Endothelin receptor antagonists, iloprost, atrial natriuretic peptide, and newer therapies such as targeted renal therapy (TRT) are discussed. A working algorithm based on current evidence is proposed. No current treatment can reverse or ameliorate CIN once it occurs, but prophylaxis is possible.

## INTRODUCTION

Imaging is an integral component of a urological evaluation. Inaccessibility of most urogenital organs to clinical examination and the complex pathophysiological disorders affecting structural and functional aspects necessitate imaging in most patients. Though several imaging modalities are available, intravenous contrast agents have a distinct role - to study precise anatomical delineation, vascularity, and to assess the function of the renal unit. Nephrotoxicity associated with intravascular contrast agents is a known entity. Though the toxicity has not been completely overcome, newly available contrast media have a better safety and efficacy profile.

The incidence of hospital acquired renal failure is on the rise and contrast induced nephropathy (CIN) is the third most common cause of hospital-acquired renal failure after hypoperfusion-induced and drug-induced renal failure. CIN contributes to approximately 11% of hospital acquired renal failure.[1] The development of contrast nephropathy not only increases the length of the hospital stay, it also increases in-hospital and long-term mortality as is evident in multiple studies.[2–4]

## CIN IN UROLOGICAL IMAGING

Most of the available literature regarding CIN follows intra-arterial administration of contrast media (CM) for angiography. Commonly performed urological investigations including intravenous pyelogram, contrast enhanced computed tomography (CT), multislice CT urography, and CT angiography involve intravenous contrast administration. In patients with and without pre-existing renal damage, earlier studies on patients who underwent intravenous urography reported CIN in 55% and 15% of the cases, respectively.[5] With low osmolar contrast medium (LOCM) for IVP, none of the patients experienced a CM related increase in serum creatinine.[6,7] In a recent study comparing iodixanol and ioversol for IVP, one out of 25 patients in each arm developed contrast nephropathy.[8] A comparison between iodixanol and iomeprol for abdominal CT revealed a significantly lower incidence of CIN with iomeprol as compared with iodixanol (6.9% *vs.* 0%).[9] Renal dysfunction was less common following intravenous route as compared with intra-arterial administration.[10] Nevertheless, while the incidence of CIN after intravenous administration of CM may be half of that seen in similar patients at high risk who receive intra-arterial CM, it should be remembered that intravenous CM enhanced examinations are performed 10 to 20-fold more often than intra-arterial ones.[11]

## DEFINITION

The most widely accepted definition of CIN is that of the European Society of Urogenital Radiology, defined as an increase in serum creatinine by >25% or 44.2 mmol/L [0.5 mg/dL]) within 3 days after intravascular administration of contrast medium, without an alternative etiology.[12] The incidence of CIN as quoted in the literature varies from 1.3% to 14.5% in view of the different criteria used to describe CIN.[13] The incidence also varies with the presence or absence of baseline renal impairment. It varies from 0–10% in patients with normal renal function and may be as high as 25–50% in patients with preexisting renal impairment or certain risk factors [Table 1].[14,15]

**Table 1:** Risk factors for CIN

Non modifiable Risk factors	Modifiable Risk factors
Age > 75 years	Per procedural volume depletion
Pre existing renal impairment	IABP use in the setting of PCI
Diabetes Mellitus	Volume of contrast used
Ejection fraction less than 40%	Concomitant use of non steroidal anti-inflammatory drugs
Hypotension/Shock	Type of contrast agent
	Recent contrast use

## PATHOPHYSIOLOGY OF CONTRAST INDUCED NEPHROPATHY

The pathogenesis of contrast-medium induced nephropathy in humans is not clear. In vitro studies and studies in animals suggest a combination of toxic injury to the renal tubules and ischemic injury partly mediated by reactive oxygen species.[16,17] Contrast media produces prolonged vasoconstriction and medullary ischemia.[18,19] Low blood flow in the medulla, which has a high demand for oxygen, might result from increased perivascular hydrostatic pressure, high viscosity, or changes in vasoactive substances such as endothelin, nitric oxide, and adenosine.[16,20] Delivery of a large osmotic load to the juxtaglomerular apparatus causes tubuloglomerular feedback and also causes disruption of the physiologic balance between the vasodilator and vasoconstrictor influences in the kidney.[21]

The marked increase in proximal tubular pressure due to increased osmotic load is associated with a gradual decrease in renal blood flow and a moderate decrease in the glomerular filtration rate. Afferent vasodilation and an increase in renin release, probably associated with efferent vasoconstriction, counteracts the fall in the glomerular filtration rate. In patients who have a reduction in endogenous vasodilators like nitric oxide and prostaglandins (e.g., high-risk patients with diabetes and renal failure), the afferent vasodilation in response to the increase in proximal tubular pressure is affected, leading to a more pronounced decrease in renal blood flow and glomerular filtration rate.[22] Adenosine has been found to enhance the renal hemodynamic effects of contrast media, resulting in local renal vasoconstriction promoting development of CIN. This is the rationale for using theophyllines for the prevention of CIN.[23]

## CLINICAL PRESENTATION OF CIN

CIN most commonly manifests as a nonoliguric and asymptomatic transient decline in renal function.[24] The serum creatinine level begins to rise within 24 hrs of contrast administration, usually peaks within 3–5 days, and returns to baseline within 10–14 days.[25] The frequency of acute renal deterioration requiring hemodialysis is small and occurs in less than 1% of the patients.[26] Patients who developed Acute Renal Failure (ARF) following percutaneous coronary intervention after exposure to contrast media were found to have increased morbidity and mortality. Complications including hematoma formation, pseudoaneurysms, stroke, coma, adult respiratory syndrome, pulmonary embolism, etc. were more common in patients who developed CIN.[2] Marenzi, *et al.* reported a 40% incidence of CIN in patients with Glomerular Filtration Rate (GFR) <60 ml per minute compared with 13% in patients with GFR >60 ml per minute in patients undergoing primary angioplasty for acute myocardial infarction. These patients were also found to have increased mortality, longer hospital stays, and complicated clinical course.[27] Urinary epithelial cell casts, debris urate, and calcium oxalate crystals are nonspecific findings in CIN and are not pathognomonic of the condition.[28]

## MANAGEMENT OF CONTRAST INDUCED NEPHROPATHY

**Figure 1:**
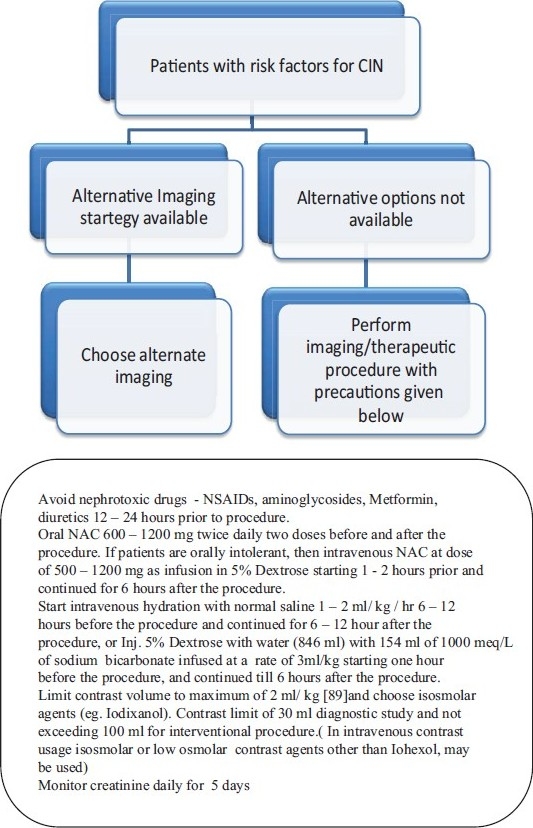
Proposed strategy for the management of Contrast Nephropathy

A simple risk score was formulated by Mehran, *et al.* for patients undergoing percutaneous coronary intervention to identify high risk subsets for focused preventive therapy and prognostication of CIN[29] [Table 2]. Alternative imaging should be done whenever possible for patients with high risk [Figure 1]. Management starts with clinical suspicion and early identification of renal function impairment as evidenced by a serial rise in creatinine levels. Serum creatinine levels should be monitored serially for 5 days after contrast exposure. Once CIN has developed, the treatment is the same as for any cause of acute renal failure, hence prevention is the key.

**Table 2:** Risk prediction after contrast exposure in patients undergoing percutaneous coronary intervention*

Risk Factor	Score	Risk Factor	Score
Hypotension	5	Se Creatinine > 1.5 mg/dl	4
IABP	5	or	
CHF	5	eGFR< 60 ml/min/1.73m2	
Age > 75	4		
Anemia	3	40 – < 60	2
Diabetes	3	20 – 39	4
Contrast Media	1 for each	< 20	6
volume	100 cc^3^		

Total risk score	Risk of Se Creatinine rise of >0.5 mg/dl or > 25% from baseline		Risk of dialysis in percentage

≤ 5	7.5		0.04
6 to 10	14		0.2
11 to 15	26.1		1.09
≥ 16	57.3		12.6

* Adapted from Mehran *et al*.[29]

Equations for calculating Creatinine clearance and GFR

Cockcroft–Gault (CandG) estimates CrCl (ml/min) $\begin{array}{l} \frac{140\left(\text{age}\right)\times \text{weight}\left(\text{kg}\right)\times 0.85\left(\text{if female}\right)}{72\times \text{Se Cr}\left(\text{mg/ml}\right)} \end{array}$ Modification of diet in renal disease (MDRD) estimates GFR (ml/min/1.73 m2) $\frac{170\times \left(\text{Se Cr}\times \text{0. 011}\right)-0.999\times \left(\text{age}\right)-0.176\times \left(\text{Se Ur}\times \text{2.801}\right)-0.170}{\left(\text{S Alb}\times \text{0.1}\right)0.318\times 1.180\left(\text{if black}\right)\times 0.762\left(\text{if female}\right)}$ (SCr, serum creatinine; SUr, serum urea; SAlb, serum albumin)

## PREVENTION OF CIN

Several agents have been considered for the prevention of CIN. Some are found to be useful, others showed promise in a few studies and some are obsolete. The proven or potentially beneficial agents include intravenous hydration with normal saline or sodium bicarbonate infusion, N-Acetyl cysteine (both oral and intravenous forms), isosmolar contrast agents, and theophyllines. Dopamine infusion and Frusemide were found to be deleterious. The role of agents like statins, fenoldopam, Iloprost, atrial natriuretic peptide, endothelin receptor antagonists, calcium channel blockers, and hemofiltration has been debated and is of questionable benefit.

### Role of hydration

Hydration with intravenous normal saline has consistently been shown to prevent or ameliorate contrast induced renal impairment in patients with and without existing renal disfunction.[30–32] Hydration prevents CIN by increasing the glomerular filtration rate by plasma volume expansion and suppressing renin–angiotensin system, thereby down regulating the tubulo-glomerular feed back mechanism.[33] Iodinated contrast agents increase urine flow and osmolar clearance resulting in a prolonged dehydrated state.[34] Hydration prevents CIN by ameliorating the effect of contrast agents. Studies comparing hydration with 0.9% and 0.45% saline have shown normal saline to be superior to half-normal saline. The advantage of isotonic saline is thought to be due to the increased sodium load, which produced more potent intravascular volume expansion and inhibition of the renin-angiotensin pathway compared with the half–isotonic saline.[30] Recently, hydration with sodium bicarbonate was found to be better than hydration with isotonic saline or a combination of isotonic saline with oral N acetyl cysteine. The incidence of CIN was significantly lower in the sodium bicarbonate group (4.5%) compared with sodium chloride alone (13.6%, P = 0.036) and tended to be lower than in the combination group (12.5%, P =.059) The enhanced efficacy in preventing CIN with this protocol was attributed to the antioxidant free-radical scavenging properties of the sodium bicarbonate preparation. Free-radical generation is found to be more in acidic media and sodium bicarbonate by decreasing the acidification of urine reduces the generation of free radicals thus protecting the kidney from oxidant injury. In this trial, 154 ml of 1000 meq/L solution of sodium bicarbonate was added to 846 ml of 5% Dextrose, which was infused at 1 ml/kg/hr. Infusion was started 6 hours prior to the procedure and was continued until 6 hours after the procedure.[35] Another study by Merten, *et al*. also showed improved results with sodium bicarbonate compared with normal saline with CIN occurring in significantly fewer patients.[36] Currently, the preferred hydration regime would be either sodium bicarbonate solution or isotonic saline infused at 1 ml/kg/hour for a minimum period of 12 – 24 hours starting at least 6 hours prior to the procedure.

### N- Acetyl Cysteine

N- Acetyl cysteine (NAC) is a thiol- containing antioxidant that increases the reducing capacity of the cell. The protective effects of this drug are due to its free-radical scavenging effect and ability to form reactive sulfhydryl compounds. It combines with nitric oxide (NO) to form S-nitrosothiol, which is a stable compound with potent vasodilatory capabilities. N- Acetyl cysteine competes with superoxide radical for NO and limits the production of damaging peroxinitrite radical. NAC has also been reported to block the expression of vascular cell adhesion molecule-1 and the activation of nuclear factor- κβ in glomerular mesangial cells.[37,38] NAC is also thought to increase the expression of NO synthase, thus enhancing endothelium dependent vasodilation and thereby increasing renal blood flow.[39] Studies have been conducted both with oral and intravenous NAC preparations and favorable effects are seen with both. Intravenous preparations is used when the time to procedure is short since the oral preparations needs to be started 12–24 hours prior to contrast administration and also in situations were oral administration is not possible. Oral preparations are preferred in situations where congestive cardiac failure limits the extent of hydration. Studies with intravenous NAC preparations have shown consistent benefit though the number of studies is less compared with the number of studies with oral NAC.[40]

Multiple meta-analyses have been done to assess the efficacy of NAC in CIN. In their meta-analysis, Alonso, *et al*. included 8 RCTs of which three showed significant benefit with NAC. The overall relative risk for CIN with the use of NAC was 0.41 (95% CI, 0.22 to 0.79; P = 0.007). The main limitation of this meta-analysis was that the studies included in this meta-analysis were heterogeneous with respect to patient population, definition of CIN, and type of radiological intervention. The degree of renal impairment was not uniform and a majority of the studies used oral NAC at varying doses.[41] In a systematic review by Bagshaw and Ghali, which included 14 randomized controlled trials, the overall pooled odds ratio for development of CIN using random-effects model was 0.54 (95% CI, 0.32–0.91, p = 0.022), suggesting a significant reduction in CIN with NAC. However, similar to the meta-analysis by Alonso, *et al*., there was considerable heterogeneity in the included studies. The authors concluded that though the overall outcome seemed promising, the efficacy of NAC was not conclusively proven.[42] In another meta-analysis of drugs used to prevent CIN by Kelly, *et al*., NAC significantly decreased the risk for contrast-induced nephropathy when compared with standard hydration (relative risk, 0.62 [95% CI, 0.44 to 0.88]). This meta-analysis included 30 studies involving NAC in the prevention of CIN and included both oral and IV preparations along with standard hydration protocols. In this meta-analysis, NAC was found to be the most effective agent to prevent CIN. The relative lack of adverse effects and inexpensiveness add to the value of this drug. The enhanced efficacy may partly be due to the outcome of weighing in by published studies with reportedly favorable outcomes with the drug, due to publication bias inherent with all meta-analysis. All included trials looked at CIN as the primary outcome; clinical endpoints of in-hospital mortality, morbidity, and dialysis dependency as a consequence of CIN were not measured.[43] The recommended dosage for NAC schedules include 600 mg–1200 mg twice daily 12–24 hours prior to procedure and continued until 12–24 hours after the procedure, along with hydration. For intravenous preparations, the dosages vary from 500 mg to 1200 mg prior to procedure followed by oral 600 mg–1200 mg until 12–24 hours after the procedure.[40,44]

### Adenosine antagonists

The most studied drug in this group is theophylline. Theophylline, a nonspecific adenosine receptor antagonist, was effective in animal studies and prevented the decline of the glomerular filtration rate after contrast injection. Enhanced adenosine triphosphate hydrolysis along with tubuloglomerular feedback results in an increase in renal adenosine concentrations. Adenosine has been found to enhance the renal hemodynamic effects of contrast media, resulting in local renal vasoconstriction.[23] In animal experiments, Arakawa had shown that in patients with impaired renal function Adenosine A1 receptors produce sustained aggravation of hemodynamics on exposure to contrast medium and theophylline, a non selective blocker of A1 and A2 receptors, causing amelioration of its effect.[45] Huber, *et al*. in two separate studies had demonstrated the benefit of theophylline in prevention of CIN in patients with chronic kidney disease and in patients admitted to the intensive care unit with at least one risk factor.[46,47] However, in a meta-analysis involving 6 RCTs evaluating the effects of theophylline on the prevention of CIN, though there was a favorable trend, the difference was not statistically significant (relative risk, 0.49 [CI, 0.23 to 1.06]). Hence, theophylline cannot be strongly recommended for the prevention of CIN. Among the various studies involving theophylline, the dose that showed maximal benefit was 200 mg twice daily 24 hours before and 48 hours after the procedure.[48]

### Contrast agents and risk of CIN

Data from animal studies have suggested that nonionic contrast media are less nephrotoxic than ionic contrast media.[49,50] Characteristics of commonly used contrast agents are summarized in Table 3. Nonionic (low-osmolality) contrast media have become increasingly popular for radiographic procedures requiring intravascular contrast because they are associated with lesser systemic and organ toxicity compared with conventional ionic (high-osmolality) contrast media.[51] In their study comparing ionic contrast diatrizoate with low osmolar agent iohexol, Rudnick, *et al*. found that iohexol was associated with significantly less nephrotoxicity than the ionic contrast agent diatrizoate in high-risk azotemic patients undergoing elective cardiac angiography. However, in non-azotemic patients, regardless of the presence or absence of diabetes mellitus, there was no evidence of reduced nephrotoxicity using nonionic agents.[52] Barrett, *et al*. in their meta-analysis found that among 25 trials comparing low osmolar contrast agents with high osmolar contrast agents the pooled odds ratio was 0.61 (95% CI; 0.48 - 0.77) in favor of low osmolar agents.[53]

**Table 3:** Classification of Contrast media based on osmolarity and ionicity

Property	High osmolar (1800 – 2100) mOsmol/kg H_2_O	Low osmolar 600 mOsmol/kg H_2_O	Low osmolar (700 – 840) mOsmol/kg H_2_O	Isosmolar 280 mOsmol/kg H_2_O
Ionicity	Ionic	Ionic	Non ionic	Non ionic
Benzene rings	Monomer	Dimer	Monomer	Dimer
Iodine to particle ratio	1.5	3	3	6
Generic names	Datrizoate	Ioxaglate	Iohexol	Iodixanol
Viscosity at 37°C	8.4	7.5	8 – 10.5	12
Nephrotoxicity	+++	++	++	++
Iodine mg/ml	370	320	350	320

In their study comparing contrast agents iohexol (low-osmolar) and iodixanol (isosmolar), Aspelin, *et al*. found the odds of nephropathy to be 11 times higher with the low osmolar contrast agent compared with isosmolar agents in high risk patients (Se creatinine 1.5 to 3.5 mg/dl) undergoing coronary angiography.[54] Iodixanol (isosmolar) was also found to be more cost effective than low osmolar iohexol in diabetic patients with impaired renal function undergoing angiography.[55] The osmotic diuresis induced by low-osmolar media is generally greater than that induced by iso-osmolar media. This diuresis may enhance distal sodium delivery increasing medullary work and inducing hypoxia or volume depletion with consequent activation of vasoregulatory hormones. If these vasoregulatory mechanisms are impaired (e.g., in patients with diabetes, renal impairment, or both), renal damage may occur after exposure to contrast mediums and this could explain the relative non toxicity of iso-osmolar contrast mediums.[56,57] Retrospective analyses have suggested that a total dose of <30 ml for diagnostic studies and <100 ml for interventional procedures lessen the risk of CIN[29] and in patients with high risk features for CIN, isosmolar agents are generally preferred. However, in patients receiving intravenous contrasts, the distinction between iso-osmolar agents and low-osmolar agents (other than iohexol) may not be as relevant.[9,58,59]

### Statins in CIN

Recent reports have suggested that statins reduce the incidence of CIN. The possible mechanism may be related to the amelioration of Angiotensin-2 mediated organ damage and increased expression of inducible nitric oxide synthase (i-NOS), which were demonstrated in animal studies.[60] In a retrospective analysis of patients with impaired renal function, patients who started on statin therapy prior to the procedure were found to have lower creatinine levels and decreased incidence of acute renal failure following cardiac catheterization.[61] Similar findings were also noted in a large registry data of 29,409 patients who underwent percutaneous coronary intervention. The incidence of CIN was 4.37% in patients who received statins compared with 5.93% who did not receive statin therapy.[62] However, in a prospective randomized two center trial of simvastatin, at a dose of 40 mg administered 12 hours prior to coronary angiography and continued 12 hours after the procedure, the incidence of CIN was not reduced compared with placebo.[63] In patients undergoing percutaneous coronary intervention, Patti, *et al*. found a lower incidence of CIN in those who were preteated with statins (3% *vs*. 27%; p = 0.0001). Long-term followup at 48 months revealed similar major cardiac events in patients who were statin-naïve without CIN and patients who developed CIN on statins.[64] In a study by Zhao, *et al*., patients undergoing primary angioplasty who were on pretreatment with statin had a lower incidence of CIN than those patients who were not receiving statin. Statin pretreatment along with anterior myocardial infarction, baseline creatinine value, time-to reperfusion, and higher contrast volume were found to be independent predictors for development of CIN.[65]

### Other agents

Agents that have been tried for prevention of CIN but not found to be useful or had deleterious outcomes include frusemide, fenoldopam, dopamine infusion, atrial natriuretic peptide, non specific endothelin receptor antagonists, prostaglandin E1, and calcium channel blockers.

### Frusemide

Dussol, *et al*. in their study comparing saline hydration, oral theophylline, and intravenous frusemide at a dose of 3 mg/kg just after the procedure found that patients receiving frusemide had a worse outcome than patients treated with saline hydration.[66]

### Fenoldopam

Fenoldopam mesylate is a specific dopamine-1 receptor agonist that produces systemic, peripheral, and renal arterial vasodilatation.[67,68] Hence, it was thought to be a favorable adjuvant in the prevention of CIN. However, four randomized controlled trials conducted with fenoldopam failed to show any added benefit compared with standard treatment strategies. The fenoldopam administration varied from 15 min to 4 hours before and 4 to 12 hours after intravenous administration of contrast media. In all studies, fenoldopam was administered as an infusion of 0.1 μg/kg^−1^ min^−1^.[69–72] In view of the above findings, fenoldopam is not used as standard therapy for the prevention of CIN.

### Dopamine

In the randomized control trial by Abizaid, *et al*. comparing hydration with 0.45% saline, aminophylline, and dopamine, the incidence of CIN was not significantly altered by the use of low-dose dopamine (2.5 mg/kg/min) beginning 2 hours before coronary angioplasty and continued for an additional 12 hours. In the same study, dopamine use after the development of CIN was found to be detrimental.[73] In another study, Diez *et al*. found no difference between plain hydration compared with the addition of dopamine at a dose of 2 mcg/kg/min starting 30 minutes prior to contrast exposure and continuing until study termination.[74] Based on the above studies, Dopamine infusion is not used for the prevention of CIN.

### Iloprost

Iloprost at a dose of 1 to 2 ng/mg/min started 30–90 min before the procedure and continued for 4 hours after the procedure was superior to placebo in preventing CIN in patients undergoing coronary angiography. However, significant hypotension was observed in patients receiving a higher dose of Iloprost.[75]

### Atrial Natriuretic Peptide

A large prospective trial conducted with atrial natriuretic peptide (ANP) failed to show any benefit compared with mannitol in decreasing the incidence of CIN. Renal blood flow was increased in both the groups but the incidence of acute renal failure was the same in both the groups. Hence, further studies are needed to define the role of atrial natriuretic peptide.[76]

### Endothelin receptor antagonist

Endothelin, a potent vasoconstrictor, was implicated in the pathogenesis of CIN. A prospective study comparing non selective endothelin receptor antagonist with placebo in patients with renal impairment undergoing coronary angiography showed an increased incidence of CIN in the group receiving the drug (56% vs. 29%; p = 0.002). The adverse finding was attributed to endothelin B receptor inhibition, which may cause vasoconstriction.[77]

### Calcium channel blockers

Three small trials of calcium channel blockers using nifedipine, amlodipine, and nitrendipine in patients with normal renal function did not show any difference between treatment groups.[78–80]

### Hemofiltration

Hemodialysis has been proposed as a prophylactic treatment for CIN owing to its ability to remove contrast media.[81–82] Marenzi, *et al*. studied the role of hemofiltration in patients with chronic kidney disease undergoing contrast exposure. There was a marked reduction in the incidence of CIN in patients undergoing hemofiltration (fluid replacement rate, 1000 ml per hour without weight loss) compared with standard saline hydration. In this study, hemofiltration was initiated 4 to 8 hours before the study and continued 18 to 24 hours after the study.[83] However, in their study where hemodialysis was done immediately after contrast exposure, Vogt, *et al*. found that the incidence of CIN was higher than that of patients treated with the standard protocol of hydration.[84] Marenzi, *et al*. compared two protocols of hemofiltration with the standard hydration regimen and found that patients who underwent hemofiltration before and after the procedure fared better than the groups allotted to either hemofiltration after procedure or standard hydration regime (control group).[85] Frank, *et al*. studied the effect of simultaneous dialysis in patients with chronic renal failure undergoing coronary angiography. The peak plasma concentration of radiocontrast medium and the incidence of CIN were not different from that of the control population.[86] Hemofiltration, though promising is invasive, costly, and not easily available. Hence, the benefits and reproducibility of the findings have to be ascertained in larger studies in multiple centers before advocating this therapy as a standard practice.

## FUTURE DIRECTIONS

Targeted renal therapy (TRT) is a novel technique where continuous infusion of intra-arterial fenoldopam into renal arteries was achieved using a specialized catheter system (Benephit™ Infusion System, FlowMedica, Inc., Fremont, CA). The Benephit System Renal Infusion Therapy (BeRITe) registry patients who were undergoing coronary angiography / coronary intervention or cardiovascular surgery received fenoldopam (285 patients out of a total of 501) infusion showed a the 71% lower incidence of CIN than was predicted (8.1% actual CIN versus 28.0% predicted; p<0.0001).[87] In a feasibility study of TRT in patients undergoing endovascular aneurysm repair (EVAR), Allie, *et al*. showed that renal function as assessed by creatinine clearance declined in only 1 patient out of total of 10 patients who had impaired renal function at baseline at 72 hours post EVAR.[88] The beneficial effect of this modality is to be confirmed in large randomized trials.

## CONCLUSION

An increase in diagnostic and interventional procedures using contrast agents contribute to a third of the cases of acute renal failure in the hospital setting. The pathogenesis of CIN in humans is still unclear. Use of risk prediction scores help in prognosticating and assessing risk of CIN in individual patients. Adequate hydration and limiting contrast volume have shown to limit the incidence of CIN. NAC and isosmolar contrast agents have been shown to be beneficial following intra-arterial administration and they may have a role in high risk individuals with intravenous administration.
